# Supplementary material for: Piper nigrum CYP719A37 Catalyzes the Decisive Methylenedioxy Bridge Formation in Piperine Biosynthesis
Source: Plants (Basel). 2021 Jan 9;10(1):128. doi: 10.3390/plants10010128 (PMC7826766; doi:10.3390/plants10010128)
Supplement: Supplementary file 1 [file plants-10-00128-s001.pdf]

Supplemental Material

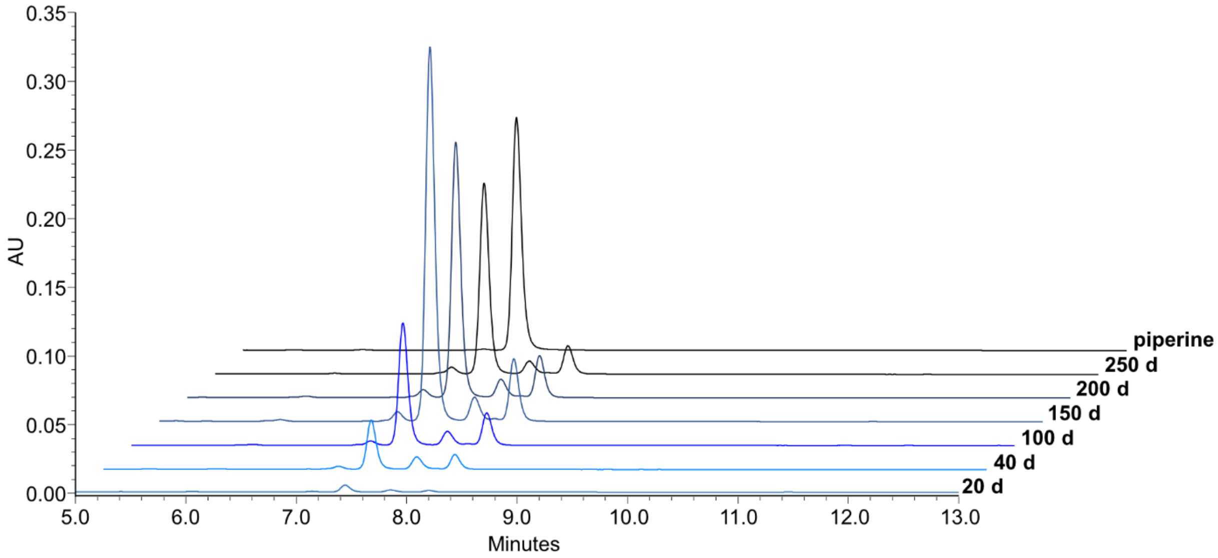

Figure S1 Accumulation of piperine during fruit development of black pepper. 5 µl of a 10 mg/ml 90% MeOH extracted fresh fruits were analysed by LC-MS and product formation recorded at 340 nm. Days post anthesis are marked.

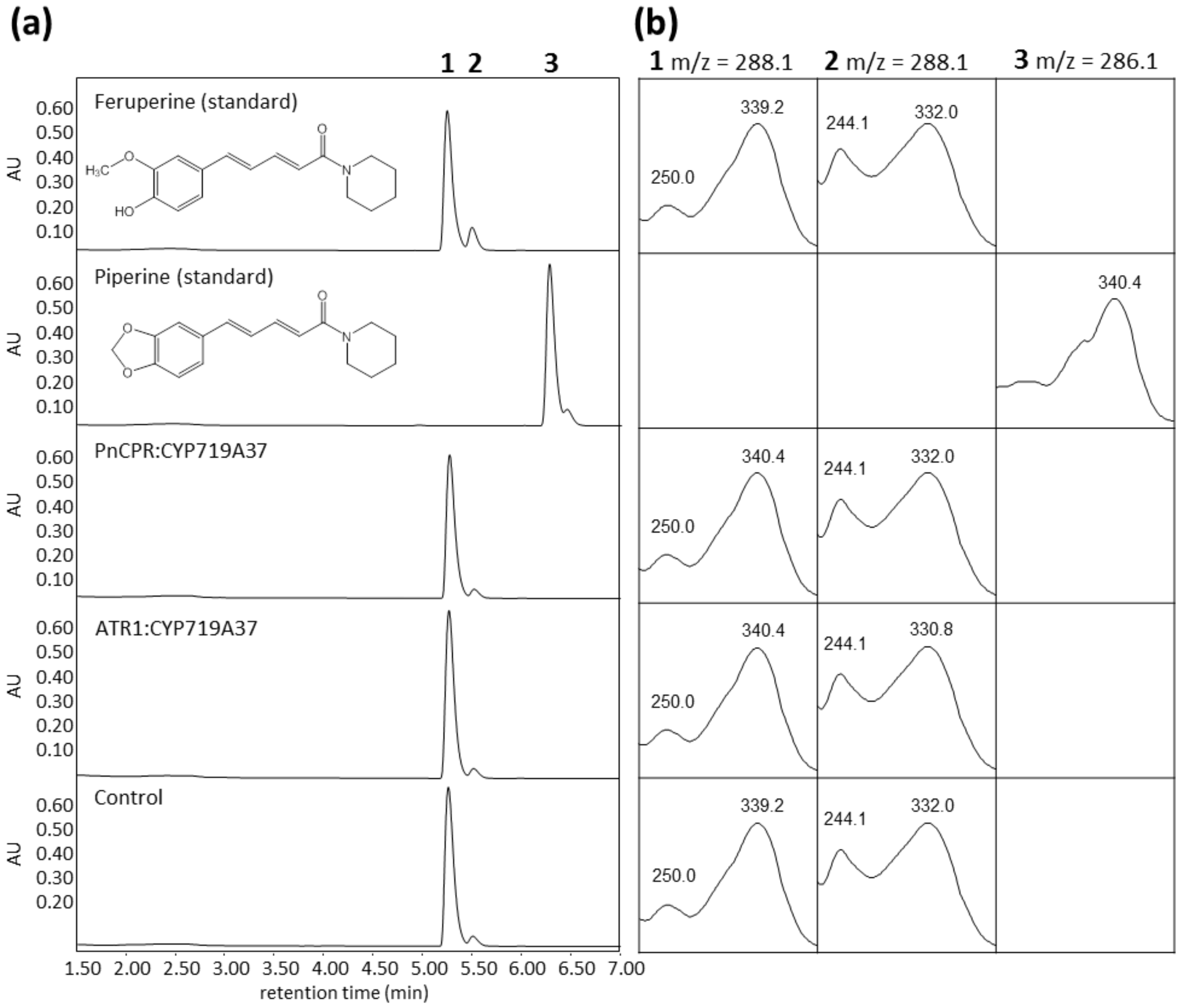

Figure S2 Microsomes from *CYP719A37*-transformed yeast were incubated with 100 µM feruperine (1) and its isomer side product (2) for up to 24 hours (shown). No product formation (piperine, 3) was observed, neither with co-transformed reductases, ATR1 nor with PnCPR1. Corresponding UV-spectra are shown on the right. Piperine and empty vector controls are also shown.

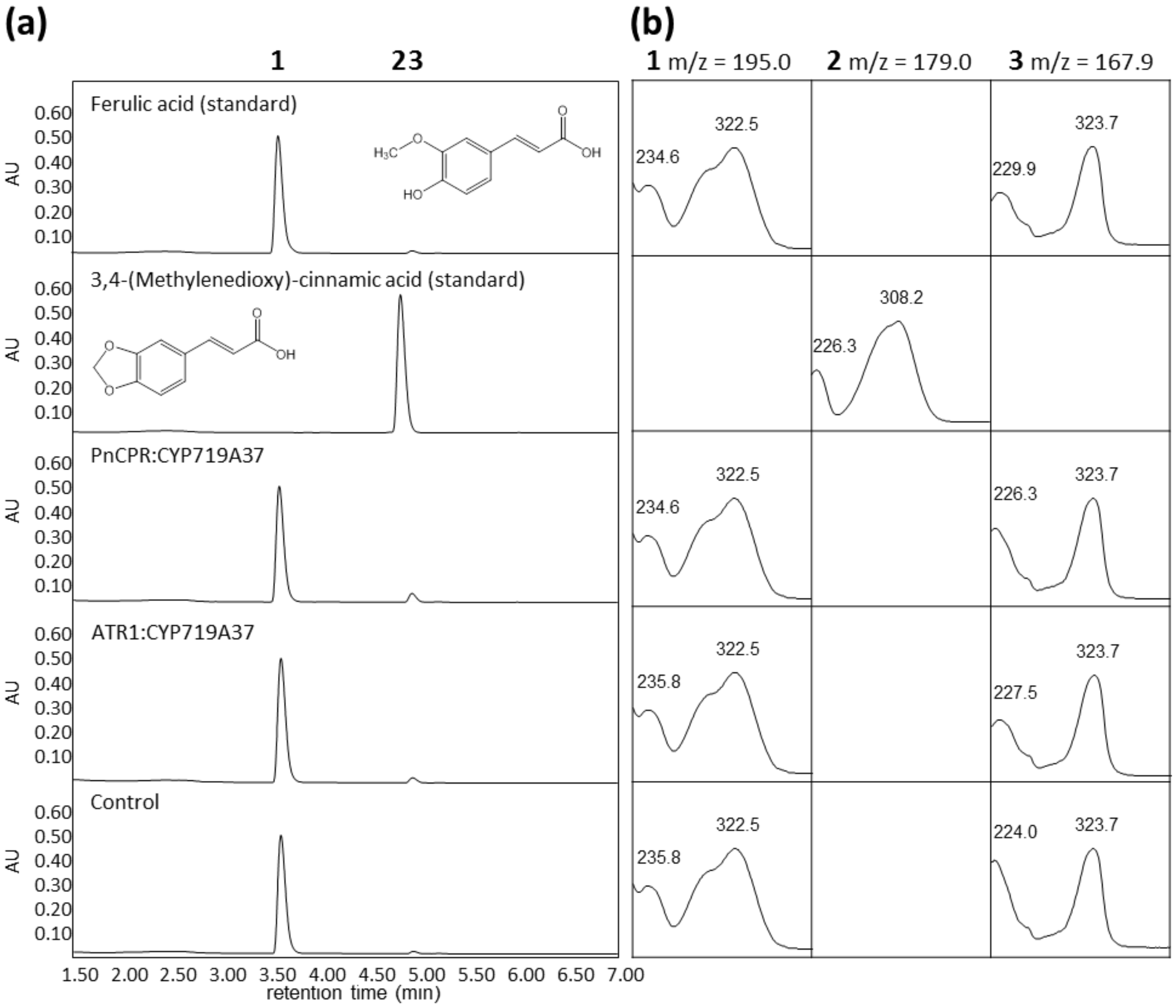

Figure S3 Microsomes from CYP719A37-transformed yeast were incubated with 100 µM ferulic acid (1) for up to 24 hours (shown). No product formation (3,4-methylendioxy cinnamic acid, 2) was observed, neither with co-transformed reductases, ATR1 nor with PnCPR1. Corresponding UV-spectra are shown on the right. The minor compound (3) is also detected in the standard and is not identical to the expected product. Ferulic acid and empty vector controls are also shown.

```
                                 membrane-anchored region
CYP719A37 (Pni) ---------M EQAQWVDPAL FSAFVSIIFF FLGMFLGRIS LGVGKGAAPR SPSSTEWPDG
CYP719A26 (Pme) ---------M EQAQWVDPTL LPAFVGIIFF FLGMFFGRSS LGAGKGAAPR STSSTEWPDG
CYP719A1  (Cja) ------MEMN PLLVCATVAI VFATTTIIRI LF-------- -------SSS SLPQMKWPSG
CYP719A5  (Eca) ---------M EESLWVVTAT VVVVFAIAKL LK-------- -------KSS SISTMEWPKG
pCYP77A2  (Atr) ---------- -MALLYLFSI LSALLFCYLF LS-------- --------SR KTKALKLPPG
CYP77A4    Ath) MFPLISFSPT SLDFTFFAII ISGFVFIITR WN-------- --------SN SKKRLNLPPG
                                      .                             .  .    : * *

CYP719A37 (Pni) PPKLPIIGNL HQLNKGGELV HHKLAKLAQS YDRAMTIWVG SWGPMIVVSD ADLAWEVLVT
CYP719A26 (Pme) PPKLPIIGNL HQLNKGGELV HHNLAKLAQS YDRAMTIWVG SWGPMIVVSD ADLAWEVLVT
CYP719A1  (Cja) PRKLPIIGNL HQLG--DDVL HVALAKLAKV HGSVMTIWIG SWRPVIVISD IEKAWEVLVN
CYP719A5  (Eca) PKKLPIIGNL HQLG--GEAF HVVLANLAKI HGTVMTIWVG AWRPMIVISD IDKAWEVLVN
pCYP77A2  (Atr) PKGWPLVGNL LQIARSGKPF VFIVNELSQK YGPIFTLKMG AR-TLIVISS AELAHEALIQ
CYP77A4    Ath) PPGWPVVGNL FQFARSGKPF FEYAEDLKKT YGPIFTLRMG TR-TMIILSD ATLVHEALIQ
                *   *::***  *:   .. .      .* :  :.  :*: :* :  .:*::*.    . *.*:

CYP719A37 (Pni) KSPDFAGRVL SKLS-HLFNA DYNTVVAYDA GPQWQSLRRG LQHGPLGPAH VSAQARFHEE
CYP719A26 (Pme) KSPDFAGRVL SKLS-HLFNA NYNTVVAYDA GPQWQSLRRG LQHGPLGPAH VSAQARFHEE
CYP719A1  (Cja) KSADYGARDM PEIT-KIASA SWHTISTSDA GSFWQNVRKG LQSGAMGPLN VAAQNQYQER
CYP719A5  (Eca) KSSDYAGRDF PEIT-KIISA NWKNISCSDS GPFWQNLRKG LQGGALAPLN VISQYQLQER
pCYP77A2  (Atr) KGQTFANRPA ETPTRAIFSS HKKTVNSAEY GPLWRSLRRN MVQGMLTPAK LRAFRPIRRK
CYP77A4    Ath) RGALFASRPA ENPTRTIFSC NKFTVNAAKY GPVWRSLRRN MVQNMLSSTR LKEFGKLRQS
                :.  :. *      :  : ..    .:   .  *. *:.:*:. :  . : . . :      :.

CYP719A37 (Pni) DMKLLVSDMM RAAKKGGNNG VVEPLAYVRR ATIRFLSRLC FGEAFNDEAF VEGMDEAVEE
CYP719A26 (Pme) DMKLLVSDMM RAAQKGGSNG VVEPLAYVRR ATIRFLSRLC FGEAFNDEAF VEGMDEAVEE
CYP719A1  (Cja) DMKRLIKAMS DEAAN--NNG IVKPLDHIKK NTVRLLTRLI FGQAFDDNKF IESMHYEIED
CYP719A5  (Eca) DMKNLITSMQ EKASK--NNG ILKPLDYLKE ETIRLLSRLI FGQSFNDENF VKGVHLALDD
pCYP77A2  (Atr) ALDKLVERLA AESKS----G LVRVLSNCRF TVFRILLCMC FGVEMDENMI EKVDSVLKKV
CYP77A4    Ath) AMDKLIERIK SEARDN--DG LIWVLKNARF AAFCILLEMC FGIEMDEETI EKMDEILKTV
                 :. *:  :   : .    *: :  *   :   .. :*  : * *  :::: :  :

CYP719A37 (Pni) TIRATG---- HARILDAFYF TRHLPIIRRS FMNTVAAKKK IESLVRPLLS ----RPAPPG
CYP719A26 (Pme) TIGATG---- HARILDAFYF TRHLPIIRRS FIDTVNAKKK IESLVRPLLS ----RPAPPG
CYP719A1  (Cja) IIRISG---- YARLAEAFYY AKYLPSHKKA EREAFLVKCR VEELVRPLLS ----SKPPTN
CYP719A5  (Eca) LVRISG---- YASLADAFKF CENLPSHKKS IREVHEVNER VVNLVKPYLV ----KNPPTN
pCYP77A2  (Atr) LITLEPRIDD FLPILQAIFV KKVAHAKHVR KEQLETLLPL IRSRKKTIQN ----AMSAEP
CYP77A4    Ath) LMTVDPRIDD YLPILAPFFS KERKRALEVR REQVDYVVGV IERRRRAIQN PGSDKTASSF
                 :         .  :  .:    .     .     :        :    :.          ..

CYP719A37 (Pni) SYLHFLLSTD AP-------E SMIIFRIFEV YLLGVDSTAS TTTWALAFLV SNQQAQEKLH
CYP719A26 (Pme) SYLHFLLSTD AP-------E NMIIFRIFEV YLLGVDSTAS TTTWALAFLV SNQQAQEKLH
CYP719A1  (Cja) SYLYFLLSQN FE-------E EVIIFCIFEL YLLGVDSTSS TTTWALAYLI REQGAQEKLY
CYP719A5  (Eca) TYLYFLNSQK FS-------D EVIISAVLEV YDLGVDSTAS TAVWALTFLV REPRVQEKLY
pCYP77A2  (Atr) AYIDSLFDLD MG--QGPPSE DMLVTLCAEF LNGGTDTTAT AVEWAMAHLV ADSEIQTRVR
CYP77A4    Ath) SYLDTLFDLK IEGRKTTPSN EELVTLCSEF LNGGTDTTGT AIEWGIAQLI ANPEIQSRLY
                :*:  * . .          : . ::    *.    *.*:*.: :  *.:: *:  :   * ::
                                          K helix
CYP719A37 (Pni) NELAQYCASQ NNQNIKAEDV GKLSYLLGVV KETMRMKPIA PLAVPHKTLK ETMLDGKRVA
CYP719A26 (Pme) NELAQYCASQ NNQIIKADDV GKLSYLLGVV KETMRMKPIA PLAVPHKTLK ETMLDGKRVA
CYP719A1  (Cja) QDIRMTLGDV D--LVKIEDV NKLKYLQGVV KETMRMKPIA PLAIPHKTAK ETTLMGTKVA
CYP719A5  (Eca) KEIIDLTGGE R--SVKVEDV SKLPYLQAVM KETMRMKPIA PMAIPHKTSR DTSLMGKKVN
pCYP77A2  (Atr) AEIDR-VSPD H--RVNDEDV EKMEYLQAFV KETLRKHPPT YFLLTHAVTE KSTLGGYDIP
CYP77A4    Ath) DEIKSTVGDD R--RVDEKDV DKMVFLQAFV KELLRKHPPT YFSLTHAVME TTTLAGYDIP
                 ::    .       :. .**  *: :* ..: ** :* :* :  : :.* . .  : * *  :
                                aromatic region                           heme-
CYP719A37 (Pni) AGTTVVVNLY AVHYNPKLWP EPEQFRPERF VIGASGGNGG GSSEYMLQSY LPFGGGMRAC
CYP719A26 (Pme) AGTTVVVNLY AVHYNPKLWP EPEQFRPERF VVGASGGNGG GSSEYMLQSY LPFGGGMRSC
CYP719A1  (Cja) KGTRIMVNLY ALHHNQNIWP DPYKFMPERF LEG---ETGT AYNKAMEQSF LPFSAGMRIC
CYP719A5  (Eca) KGTSIMVNLY AIHHNPKVFP EPYKFIPERF LQGQ--ESKY GDIKEMEQSL LPFSAGMRIC
pCYP77A2  (Atr) EGVNVDFYTP SISDDPKLWG DPERFRPERF VDKMDGDDDA DLTGVKGVKM MPFGVGRRIC
CYP77A4    Ath) AGVNVEVYLP GISEDPRIWN NPKKFDPDRF MLGKE---DA DITGISGVKM IPFGVGRRIC
                 *. : .    .:  : .::  :* :* *:** :                  .  :**. * * *
                  binding domain
CYP719A37 (Pni) AGMEVGKLQV AMVVANLVMS FKWLPEAEGK MPDLAEDMTF VLMMKKPLAA KIVPRA--
CYP719A26 (Pme) AGMEVGKLQV AMVVANLVMA FKWLPEEEGK MPDLAEDMTF VLMMKKPLAA KIVPRA--
CYP719A1  (Cja) AGMDLGKLQF AFALANLVNA FKWSCVEEGK LPDMGEELSF VLLMKTPLEA RIAGRNV-
CYP719A5  (Eca) AGMELGKLQY GFSLASLVEA FKWTCAVDGK LPDLSEDHCF ILLMKNPLEA RITPRTQL
pCYP77A2  (Atr) PGLGLGMLHI SLMVATMVQQ FEWLPNPAVP RVDMSEKFEF TVLMKTPLLA IAQPRKK-
CYP77A4    Ath) PGLAMATIHV HLMLARMVQE FEWCAHPPGS EIDFAGKLEF TVVMKNPLRA MVKPRI--
                .*: :. ::   : :* :*   *:*          *:. .  *  ::**.** *     *
```

Figure S4 Amino acid sequence of CYP719A37 (MT643912). Characteristic features and relevant amino acids are shown.
